# Association of body mass index and visceral fat with heart rate variability in university students: a BMI-stratified analysis

**DOI:** 10.3389/fphys.2026.1830583

**Published:** 2026-05-29

**Authors:** Yanfeng Li, Jiayi Li, Ruixue Zhao, Jiameng Wang

**Affiliations:** 1 School of Sports Training, Chengdu Sport University, Chengdu, Sichuan, China; 2 Faculty of Physical Education, Yan’an University, Yan’an, Shaanxi, China; 3 Yan’an University, Yan’an, Shaanxi, China; 4 Hainan Province Key Laboratory of Sports and Health Promotion, Hainan Medical University, Haikou, Hainan, China

**Keywords:** body mass index, cardiovascular disease risk, obesity, PPG-derived pulse rate variability (PRV), visceral fat index (VFI)

## Abstract

**Objective:**

This study aims to investigate the relationship between obesity-related metrics and resting photoplethysmography (PPG)-derived pulse rate variability (PRV) parameters among university students, with a particular focus on variations across BMI categories, patterns of fat distribution, and sex differences.

**Methods:**

In this cross-sectional investigation, a total of 1, 556 students from Yan’an University were categorized into underweight, normal weight, overweight, and obese groups based on BMI criteria. Body composition was evaluated using multi-frequency bioelectrical impedance analysis, measuring parameters such as body fat percentage, skeletal muscle mass, waist-to-hip ratio (WHR), visceral fat index, and fatty liver risk index. Resting heart rate and pulse rate variability (PRV) metrics were recorded through a photoplethysmography-based device. Differences among groups were assessed using one-way ANOVA, while associations were explored using Pearson correlation and BMI-stratified multivariable linear regression models, supported by bootstrap validation with 5, 000 resamples.

**Results:**

Body composition parameters showed significant variation across BMI groups (all P< 0.001). Relative to underweight and normal-weight individuals, those in the overweight and obese categories demonstrated a less favorable resting autonomic pattern, primarily reflected by elevated resting heart rate and reduced Standard Deviation of Normal-to-Normal (SDNN). Among males, SDNN values were lower in the obese group compared to those with normal weight (48.63 ± 16.83 vs. 60.46 ± 15.00 ms). In females, obesity was linked to decreased SDNN (39.74 ± 12.52 vs. 55.06 ± 15.77 ms), increased resting heart rate (89.75 ± 8.71 vs. 80.82 ± 9.40 bpm), and a reduced PRV index (10.94 ± 3.72 vs. 14.84 ± 4.44). Certain normalized frequency-domain measures did not demonstrate consistent differences across stratified groups. Within the obese category, SDNN exhibited inverse correlations with WHR, visceral fat index, and fatty liver risk index. Additionally, across all BMI categories, resting heart rate showed an independent negative association with both SDNN and PRV index.

**Conclusion:**

In university students, overweight and obesity were linked to poorer resting autonomic function. Measures such as SDNN and PRV index appear to provide greater sensitivity than certain normalized frequency-domain parameters in detecting early obesity-related autonomic dysfunction in young adults.

## Introduction

Obesity has emerged as a significant global public health concern, with steadily increasing prevalence across children, adolescents, and adults (Bhatti et al., 2020; Chen et al., 2023). It is well established as a key risk factor for conditions such as diabetes, hypertension, cardiovascular diseases (CVDs), and visceral fat accumulation, making it a major driver of cardiometabolic impairment (Perumpail et al., 2017; Karim et al., 2022; Nie et al., 2024). In addition to these metabolic and cardiovascular effects, accumulating evidence indicates that obesity is strongly linked to autonomic nervous system (ANS) dysfunction, which may serve as an early mechanistic pathway connecting excess adiposity to heightened cardiovascular risk (Prado et al., 2010; Head et al., 2014; Lim et al., 2015).

Heart rate variability (HRV), which represents the beat-to-beat variation in sinus rhythm, is a simple, noninvasive, and cost-effective measure used to evaluate cardiac autonomic regulation (Catai et al., 2020; Young and Benton, 2018; Sendeski et al., 2024). Lower HRV is typically associated with reduced parasympathetic (vagal) activity, relative sympathetic dominance, and diminished cardiovascular adaptability. As a result, it is widely considered an early indicator of increased cardiovascular risk (Prasad et al., 2018; Jabbour and Iancu, 2021; Ba., 2024). Previous research across various age groups has consistently reported that higher BMI and increased adiposity are linked to elevated resting heart rate and decreased HRV. These findings reinforce the idea that excess body fat, particularly central and visceral fat accumulation, is associated with an impaired resting autonomic profile (Speer et al., 2021; Sjoberg et al., 2011; Skrapari et al., 2012; Baqar et al., 2019; Petretta et al., 1995; Gutin et al., 2005; Tian et al., 2015; Kono et al., 2019; Lindmark et al., 2012; Janzen et al., 2023; Verma et al., 2024; Yadav et al., 2017). From a mechanistic perspective, a likely explanation is that progressive fat accumulation, especially visceral fat, is often associated with insulin resistance, chronic low-grade inflammation, disrupted hormonal signaling, heightened sympathetic nervous system activity related to stress, and impaired baroreflex-mediated vagal control. Together, these factors may lead to reduced parasympathetic influence and a decrease in overall variability of normal-to-normal (NN) intervals. However, it is important to emphasize that this explanation represents a biologically plausible interpretation supported by existing literature, rather than direct evidence of causal relationships.

Nevertheless, a number of important issues remain inadequately addressed. First, the current body of evidence is highly heterogeneous, including studies involving children, adolescents, adults, and diverse ethnic and national populations, while data specifically focused on university students are still relatively scarce. First-year university students were not chosen because they constitute a clearly defined high-risk clinical group, but rather because they represent an early, predominantly preclinical stage of adulthood in which obesity-related autonomic changes may already be emerging prior to the onset of overt cardiometabolic disease. This makes them a particularly valuable group for early detection and preventive efforts. Second, not all HRV parameters appear to be equally effective in identifying early obesity-related autonomic alterations. In young, otherwise healthy populations, such changes may initially present as a general reduction in overall sinus rhythm variability rather than a distinct shift in normalized frequency-domain measures. As a result, indices that capture overall variability, such as SDNN and the HRV triangular index (HRV index), may demonstrate greater sensitivity than certain normalized frequency-domain parameters in detecting early autonomic dysregulation associated with obesity (Jabbour and Iancu, 2021; Sendeski et al., 2024; Farah et al., 2013). Third, the influence of sex on the relationship between obesity and HRV is still not fully understood, although differences in fat distribution, hormonal milieu, and stress-response mechanisms likely contribute to sex-specific patterns of autonomic regulation (Sheema and Malipatil, 2015; Soumya et al., 2022; Fu and Ogoh, 2019; Gaal et al., 2006; Gao and Ma, 2022; Greenberg et al., 2022; Lazarova et al., 2009).

Against this background, the present study examined the relationships between obesity-related measures and resting HRV parameters in a cohort of 1, 556 first-year university students, with specific focus on differences across BMI categories, patterns of fat distribution, and sex-related variations. The study also explored whether global variability measures, including SDNN and HRVindex, could serve as useful indicators for detecting early obesity-related autonomic dysfunction in young adults. By identifying HRV markers with greater sensitivity in this relatively young, preclinical population, the study seeks to support early cardiovascular risk assessment and cost-effective health monitoring strategies among university students.

## Methods

### Study subjects

As of September 1, 2023, the study population consisted of students enrolled across 18 departments at Yan’an University. A stratified sampling method was employed to recruit 1, 600 students, ensuring adequate representation from different academic departments and regions of origin. Participants were classified based on body mass index (BMI) into four groups: underweight (BMI< 18.5 kg/m²), normal weight (18.5 kg/m² ≤ BMI< 24 kg/m²), overweight (24 kg/m² ≤ BMI< 28 kg/m²), and obese (BMI ≥ 28 kg/m²).

### Inclusion criteria

Students registered with the university student management center and officially enrolled as of September 1, 2023. Students who voluntarily agreed to participate and provided written informed consent.

### Exclusion criteria

Participants with a known history of congenital heart disease, liver disease, or other medical conditions that could potentially influence the study outcomes were excluded. Prior to data collection, all participants underwent a physical examination and relevant clinical screening at the Affiliated Hospital of Yan’an University in Yan’an, Shaanxi Province, under the supervision of qualified medical personnel. Individuals diagnosed with abnormal lipid metabolism, cardiovascular disorders, fatty liver disease, or any other conditions that might affect the results were also excluded. To reduce potential confounding effects, participants were instructed to avoid alcohol and other stimulants for 24 hours before testing.

This study received ethical approval from the Ethics Committee of Yan’an University (Approval No. YAU-G20230101). All participants provided written informed consent prior to participation. In total, 1, 556 individuals with complete and valid data were included in the final analysis.

### Study methods

#### Basic data collection and anthropometric measurements

Baseline demographic information, including age and sex, was obtained from all participants. Height and weight were measured by trained research personnel following standardized procedures, and BMI was calculated as weight in kilograms divided by height in meters squared (kg/m²). In this study, BMI was used as an anthropometric indicator and grouping variable to represent overall weight status, rather than as a direct measure of body composition.

#### Body composition assessment

Body composition was evaluated using multi-frequency bioelectrical impedance analysis (BIA) with the InBody 770 body composition analyzer (Biospace Co., Ltd., Seoul, Korea). This system estimates body composition variables such as fat mass, skeletal muscle mass, and visceral fat-related indices by assessing electrical impedance across the trunk and limbs at multiple frequencies (Cho et al., 2022). In this study, the assessed parameters included the visceral fat index (VFI) as well as other indicators related to fat distribution. The device applies multi-frequency signals at 1, 5, 50, and 250 kHz, enhancing the accuracy of body composition measurements. However, since its predictive model is based on segmental impedance properties, results may be affected by factors such as hydration level, recent food consumption, and bladder fullness.

To reduce potential confounding effects, all participants were assessed using a standardized testing protocol. Measurements were carried out at 08:00 hours after an overnight fast, and participants were instructed to void their bladder prior to evaluation. They were also required to abstain from alcohol and other stimulants for at least 24 hours before testing. Furthermore, all procedures were performed only after completing the recommended device warm-up and quality assurance checks to ensure consistency and reliability of the measurement.

#### Measurement of autonomic nervous system–related indices

Heart rate (HR) and PPG-derived pulse rate variability (PRV) assessments were conducted by two trained evaluators following a standardized measurement protocol. Prior to testing, each participant was fully briefed on the procedures and necessary precautions. All recordings were carried out in a quiet, enclosed indoor setting designed to minimize external disturbances. To control for potential circadian influences on autonomic function, all PRV measurements were consistently performed within a defined afternoon period between 15:00 and 18:00.

After arrival in the testing environment, participants were instructed to sit quietly for a minimum of 5 minutes to allow stabilization of their baseline physiological state. PRV assessment was then performed in a seated resting position, during which participants were required to remain silent, avoid speaking, and minimize any bodily movements. PRV parameters were recorded using the Ubpulse T1 system (Biospace, Korea), with the sensor attached to the left index finger.

Since breathing patterns can significantly affect PRV measurements, participants were instructed to breathe calmly and naturally throughout the test, with respiration maintained within a range of 12–20 breaths per minute. This approach was used to limit respiratory-related interference while ensuring a relaxed physiological state. If a participant’s HR exceeded the predefined threshold of 80 beats per minute during the initial assessment, the measurement was repeated after a brief rest period to minimize the effects of anxiety or transient physiological variation. This HR threshold was applied consistently throughout the repeated-measurement procedure, with no alternative threshold used in the analysis. The final stable recording obtained under the standardized resting protocol was used for statistical analysis.

To ensure data integrity, all ectopic beats and artifacts were identified and processed using Kubios PRV analysis software (Version 4.2.0, University of Eastern Finland). The software automatically detects and corrects abnormal inter-pulse intervals arising from motion artifacts, signal disturbances, or premature beats through pulse interval detection and time-domain filtering procedures. In this study, a threshold-based artifact correction method was applied with the filter intensity set to Medium. A maximum artifact correction rate of less than 5% was considered acceptable for inclusion; recordings exceeding this limit were reviewed and, if necessary, repeated or excluded from analysis. Definitions of the PRV parameters used are presented in **Table 1**.

**Table 1:** Parameters for PPG-derived pulse rate variability indicators.

PPG-derived PRV indicator	Definition	Parameter meaning
HR/pulse rate beats/min	Average pulse rate derived from inter-pulse intervals	Represents the mean pulse rate during the recording period.
SDNN ms	Standard deviation of all normal-to-normal inter-pulse intervals	Reflects overall variability of PPG-derived pulse intervals and is associated with global autonomic modulation.
LF ms²	Low-frequency power of PRV	Represents the low-frequency component of PRV; influenced by both sympathetic and parasympathetic activity as well as baroreflex-related mechanisms.
HF ms²	High-frequency power of PRV	Represents the high-frequency component of PRV and is commonly associated with respiratory-related vagal (parasympathetic) modulation.
LFnorm n.u.	LF/(LF + HF) × 100	Represents the relative contribution of LF power to total normalized PRV power; should not be interpreted as a direct marker of sympathetic activity.
HFnorm n.u.	HF/LF + HF × 100	Represents the relative contribution of HF power to total normalized PRV power; mainly associated with vagal modulation but not a direct measurement of parasympathetic nerve activity.
LF/HF	Low-frequency power/high-frequency power	Used as a relative index of autonomic modulation; should not be interpreted as a direct measure of sympathovagal balance.
PRV-index	PPG-derived pulse rate variability index	Represents a composite PRV metric derived from PPG signals rather than ECG-derived HRV.

### Data processing methods

Data input and validation were carried out independently by two investigators to ensure accuracy. Records containing more than one-third missing information were removed from the dataset. For the remaining data, missing values were addressed through multiple imputation to minimize bias and avoid underestimating variability. Data organization was conducted using Microsoft Excel, while statistical analyses were performed with SPSS version 26.0. Categorical variables are reported as counts and corresponding percentages. The distribution of continuous variables was evaluated using the Shapiro–Wilk test, with normally distributed data presented as mean ± standard deviation (SD). Differences in body composition and PRV-related parameters across BMI categories were examined using one-way analysis of variance (ANOVA), conducted separately for male and female participants. Pearson correlation analysis was applied to explore relationships between PRV-related measures and body composition indicators within BMI-defined groups. To further identify independent associations, BMI-stratified multivariable linear regression analyses were performed, with SDNN and PRV index specified as dependent variables, while independent variables were chosen based on both correlation findings and physiological significance. Model robustness was evaluated through 5, 000 bootstrap resamples, with corresponding bootstrap estimates of the mean, standard error, and 95% confidence intervals (CIs) reported. The predictive performance of the model was assessed using adjusted R², root mean square error (RMSE), and mean absolute error (MAE). Potential multicollinearity among variables was examined using tolerance values, variance inflation factor (VIF), condition index, and variance decomposition proportions. Statistical significance was defined as a two-tailed P value less than 0.05.

## Results

### Baseline characteristics

The baseline profile of the participants is summarized in **Table 2**.

**Table 2:** Physical characteristics of the 1, 556 study participants ( $\bar{x}$ ± *s*).

Sex (N = 1556)	Participants (N = 1556)	Age (years)	Height (cm)	Weight (kg)	BMI (kg/m²)
Male (731)	A (133)	18.75 ± 0.68	175.71 ± 4.85	99.52 ± 9.16	32.21 ± 2.30
	B (175)	19.20 ± 1.09	177.03 ± 5.30	83.52 ± 7.51	26.79 ± 1.47
	C (338)	18.95 ± 0.88	177.66 ± 5.70	68.26 ± 7.16	21.59 ± 1.68
	D (85)	18.84 ± 0.75	175.81 ± 5.16	53.54 ± 4.06	17.29 ± 0.76
Female (825)	A (77)	18.83 ± 0.58	160.75 ± 5.24	84.19 ± 9.27	32.48 ± 1.93
	B (132)	18.70 ± 0.76	164.02 ± 5.43	72.92 ± 6.75	27.03 ± 1.53
	C (469)	18.84 ± 0.89	163.63 ± 5.49	56.93 ± 5.78	21.24 ± 1.70
	D (147)	18.90 ± 0.78	164.10 ± 5.41	46.89 ± 3.52	17.41 ± 0.74

Groups: A, Obese; B, Overweight; C, Normal; D, Underweight.

### Analysis of body composition and PRV indicators across BMI categories.

As shown in **Table 3**, statistically significant variables were identified in body composition measures, including body fat percentage, skeletal muscle mass, waist-to-hip ratio (WHR), visceral fat index (VFI), and fatty liver risk index, among male and female participants across different BMI categories (p<.001).

**Table 3:** Distribution of body composition indicators by BMI category among male and female university students ( $\bar{x}$ ± *s*).

Index	Sex	A (210)	B (307)	C (807)	D (232)	F	P
BFP	Male (731)	26.22 ± 2.96	19.70 ± 2.94	11.92 ± 3.65	5.42 ± 2.78	319.627	p < 0.001**
	Female (825)	39.25 ± 1.56	32.79 ± 2.23	24.53 ± 4.14	16.54 ± 2.79	288.381	p < 0.001**
SMM	Male (731)	39.85 ± 3.19	36.14 ± 3.11	32.03 ± 2.92	26.78 ± 1.93	175.616	p < 0.001**
	Female (825)	28.22 ± 3.12	25.86 ± 2.92	21.10 ± 2.53	18.04 ± 1.52	151.171	p < 0.001**
WHR	Male (731)	1.01 ± 0.05	0.91 ± 0.03	0.82 ± 0.05	0.78 ± 0.04	227.175	p < 0.001**
	Female (825)	1.06 ± 0.04	0.95 ± 0.04	0.86 ± 0.07	0.79 ± 0.03	131.621	p < 0.001**
VFI	Male (731)	11.50 ± 1.51	7.82 ± 1.49	4.34 ± 1.48	1.88 ± 1.03	373.420	p < 0.001**
	Female (825)	14.72 ± 1.00	10.87 ± 1.65	7.42 ± 1.47	4.64 ± 0.89	332.514	p < 0.001**
FLRI	Male (731)	88.33 ± 3.81	47.55 ± 23.82	4.09 ± 8.15	0.03 ± 0.01	670.497	p < 0.001**
	Female (825)	90.00 ± 0.02	80.30 ± 9.60	23.58 ± 19.00	0.05 ± 0.01	368.729	p < 0.001**

p<.001**.

Groups: A, Obese; B, Overweight; C, Normal; D, Underweight.

BFP, Body fat percentage; SMM, Skeletal muscle mass; WHR, Waist-to-hip ratio; VFI, Visceral fat index; FLRI, Fatty liver risk index.

**Figure 1:**
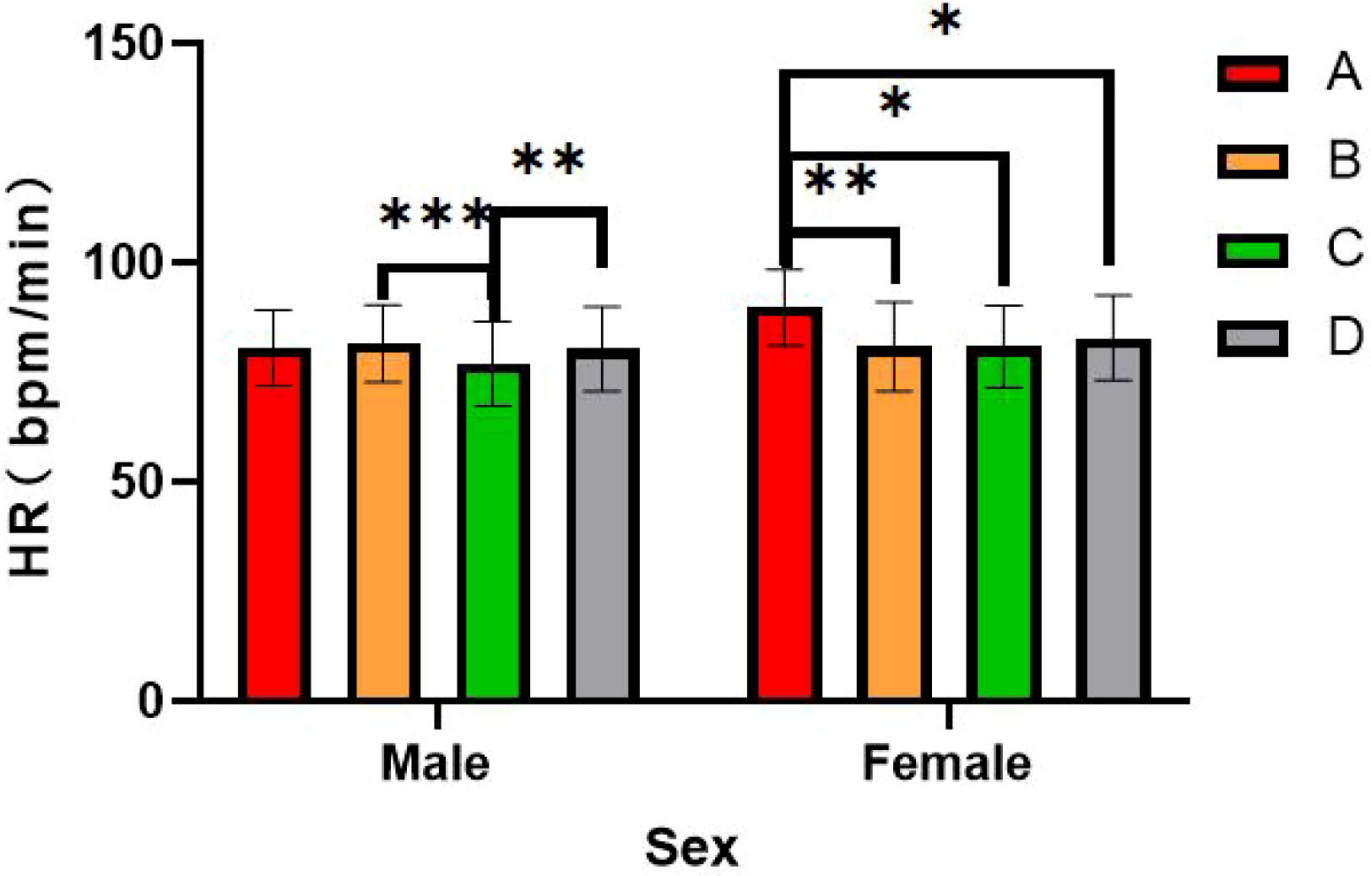
Comparison of heart rate (HR) variations across body mass index (BMI) categories among male and female university students (p<.001***;p<.001**; p<.05*).

**Figure 2:**
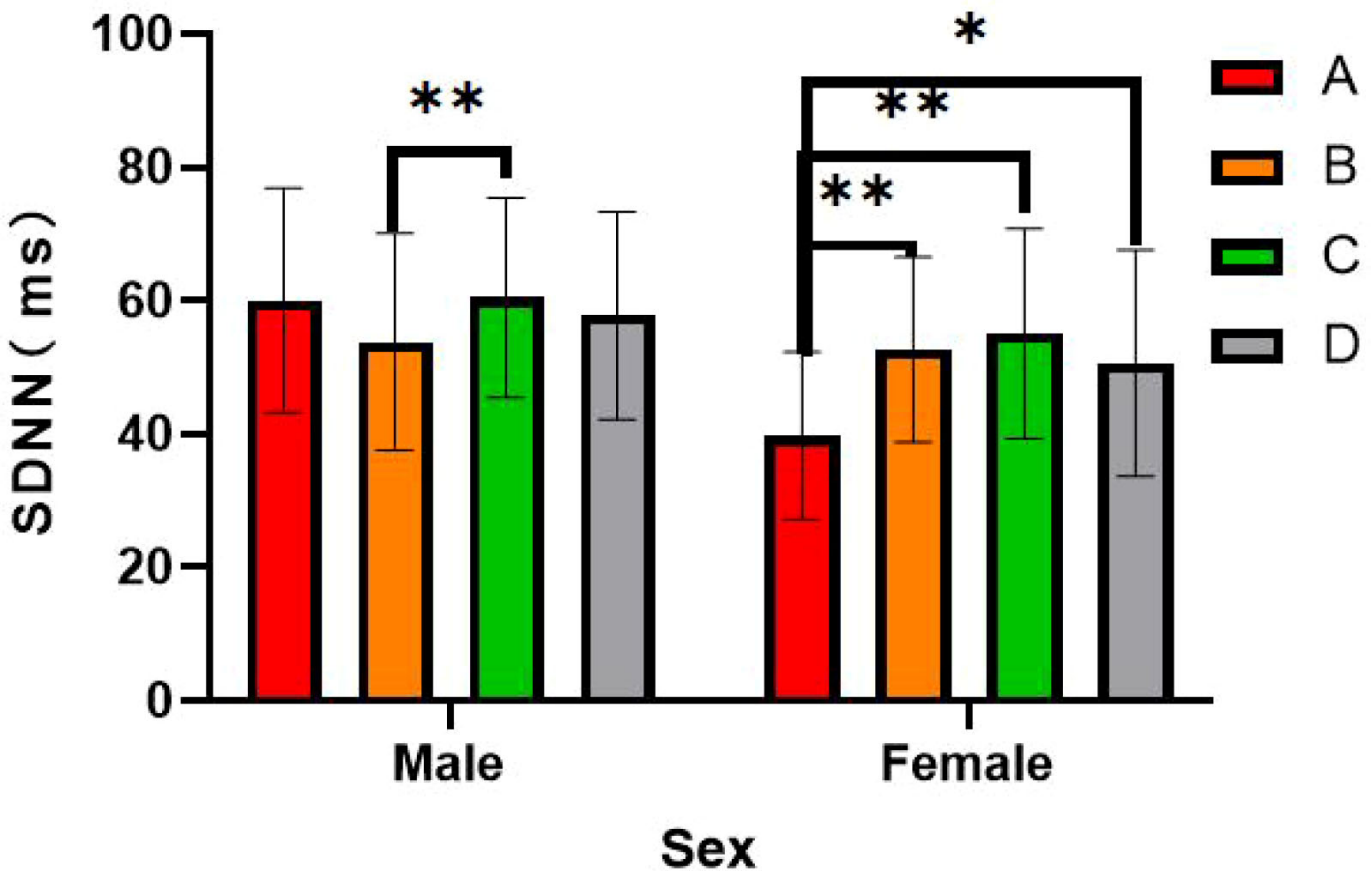
Comparison of SDNN variations across BMI categories among male and female university students (p < 0.01 **; p < 0.05 *).

**Figure 3:**
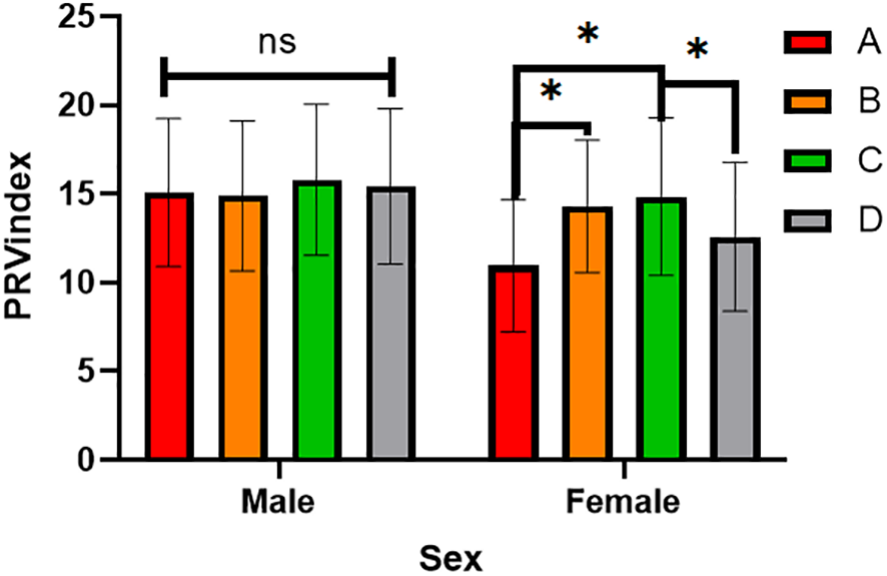
Comparison of PRV index variations across BMI categories among male and female university students (p < 0.05 *; ns, not significant).

As shown in **Table 4**, significant variations (p<.05) were found in PRV parameters, including HR and SDNN, among male and female students across BMI classifications. Additionally, low frequency (LF) values in males and the PRV index in females showed statistically significant differences (p<.05). **Figures 1**–**3** further indicate that HR is markedly elevated in both obese and overweight groups compared to normal-weight and underweight groups, with a more pronounced effect observed in females. In contrast, SDNN values are reduced in the obese and overweight categories, particularly among female participants. Moreover, the PRV index was considerably lower in obese females compared with their normal-weight and underweight counterparts.

**Table 4:** Comparison of PRV indicators across BMI categories among male and female university students ( $\bar{x}$ ± *s*).

Index	Sex	A (210)	B (307)	C (807)	D (232)	F	P
HR (bpm)	Male (731)	80.54 ± 8.65	81.58 ± 8.78	76.91 ± 9.62	80.32 ± 9.67	6.498	p < 0.001**
	Female (825)	89.75 ± 8.71	80.77 ± 10.11	80.82 ± 9.40	82.86 ± 9.72	4.220	0.006*
SDNN (ms)	Male (731)	48.63 ± 16.83	53.83 ± 16.33	60.46 ± 15.00	57.79 ± 15.61	3.732	0.011*
	Female (825)	39.74 ± 12.52	52.68 ± 13.87	55.06 ± 15.77	50.65 ± 16.96	5.180	0.002*
LF (ms^2^)	Male (731)	6.99 ± 0.68	6.81 ± 0.91	7.14 ± 0.82	7.16 ± 1.01	3.044	0.029*
	Female (825)	6.68 ± 1.36	6.84 ± 0.76	6.86 ± 0.94	7.01 ± 1.27	0.717	0.542
HF (ms^2^)	Male (731)	6.61 ± 0.98	6.28 ± 0.88	6.53 ± 0.85	6.41 ± 1.00	1.811	0.144
	Female (825)	5.88 ± 1.28	6.54 ± 0.83	6.45 ± 0.98	6.32 ± 1.22	1.830	0.141
LFnorm (n.u.)	Male (731)	51.22 ± 3.59	51.94 ± 3.30	51.94 ± 3.14	52.40 ± 3.22	0.817	0.485
	Female (825)	51.64 ± 5.67	50.62 ± 2.82	51.02 ± 3.41	51.88 ± 3.84	1.893	0.130
HFnorm (n.u.)	Male (731)	48.64 ± 3.37	47.83 ± 4.25	48.46 ± 4.90	47.39 ± 4.47	1.146	0.330
	Female (825)	46.70 ± 10.46	50.77 ± 7.29	49.14 ± 6.12	49.19 ± 7.88	1.750	0.156
PRVindex	Male (731)	15.07 ± 4.17	14.88 ± 4.23	15.80 ± 4.26	15.43 ± 4.38	1.055	0.368
	Female (825)	10.94 ± 3.72	14.31 ± 3.73	14.84 ± 4.44	12.59 ± 4.20	8.446	p< 0.001**

p<.001**; p<.05*.

Groups: A, Obese; B, Overweight; C, Normal; D, Underweight.

### Correlation analysis of body composition and PRV indicators across BMI categories

As presented in **Table 5**, in the obese group, HR, SDNN, and PRV index showed significant associations with sex, height, body fat percentage, BMI, VFI, and FLRI (p<.001). Within the overweight group, LFnorm demonstrated significant correlations with these same variables (p<.001). In the normal-weight group, HR, SDNN, and LFnorm were also significantly related to sex, height, body fat percentage, BMI, VFI, and FLRI (p<.001). Meanwhile, in the underweight group, SDNN and PRV index were significantly associated with sex, height, body fat percentage, BMI, VFI, and FLRI (p<.001).

**Table 5:** Correlation between PRV parameters and body composition indicators among university students stratified by BMI category ( $\bar{x}$ ± *s*).

Groups	Index	S	A	H	W	BFP	SM	BMI	WHR	VFI	FLRI
A (210)	HR (bpm/min)	.458**	0.200	-.424**	-0.295	.389*	-.355*	0.11	0.276	0.27	0.281
	SDNN (ms)	-.534**	0.072	.457**	0.155	-.573**	.359*	-.384*	-.557**	-.522**	-.561**
	PRVindex	-.445**	-0.021	.420*	0.158	-.463**	0.304	-.330*	-.447**	-.423*	-.443**
B (307)	LFnorm (n.u.)	-.212*	0.105	0.093	-0.005	-.222**	0.093	-0.109	-.255**	-.184*	-.225**
C (807)	HR (bpm/min)	.202**	-0.048	-.198**	-.156**	.223**	-.214**	-0.005	.156**	.216**	.173**
	SDNN (ms)	-.172**	-0.003	.094*	.095*	-.149**	.150**	0.04	-0.006	-.114**	-.090*
	LFnorm (n.u.)	-.139**	0.03	.185**	.123**	-.174**	.166**	-0.024	-.128**	-.182**	-.129**
D (232)	SDNN (ms)	-.213*	-0.064	.271**	.291**	-.232**	.282**	0.044	-.192*	-.250**	-0.148
	PRVindex	-.315**	-0.003	.237**	.274**	-.262**	.327**	0.079	-0.131	-.251**	-.178*

p<.001**; p<.05*.

S, sex; A, age; H, height; W, weight; BFP, Body fat percentage; SM, Skeletal muscle; VFI, Visceral fat index; FLRI, Fatty liver risk index.

Groups: A, Obese; B, Overweight; C, Normal; D, Underweight.

### Linear regression analysis of visceral fat index and BMI according to PRV indicators

As presented in **Table 6**, the BMI-stratified multivariable linear regression models, with SDNN as the dependent variable, demonstrated that HR consistently acted as a significant independent negative predictor across all four subgroups. Following internal validation using 5, 000 bootstrap samples, the bootstrap estimates closely matched the original regression coefficients, and all 95% confidence intervals (CIs) excluded zero, indicating strong model stability and robustness. Specifically, in the obese group (Group A, n = 210), both HR (B = −1.049, 95% CI: −1.44 to −0.68, P< 0.001) and WHR (B = −146.572, 95% CI: −218.18 to −73.38, P = 0.001) were independently and negatively associated with SDNN. In the overweight group (Group B, n = 307), HR remained the only significant independently negative predictor of SDNN (B = −0.736, 95% CI: −0.97 to −0.49, P< 0.001). In the normal-weight group (Group C, n = 807), HR (B = −0.705, P< 0.001), sex (B = −6.29, P< 0.001), and height (B = −0.26, P = 0.006) were identified as independent determinants of SDNN. In the underweight group (Group D, n = 232), HR showed a significant independent negative association with SDNN (B = −0.902, 95% CI: −1.11 to −0.69, P< 0.001), while total body water was independently and positively associated with SDNN (B = 0.639, 95% CI: 0.18 to 1.08, P = 0.008). Overall, the obese group demonstrated the strongest model fit, with the highest explanatory capacity (adjusted R² = 0.571).

**Table 6:** Multivariable linear regression analysis of SDNN across BMI categories among university students with 5, 000-sample bootstrap internal validation.

Groups	Variate	Non-standardized coefficient		Bootstrap SE	95% CI	t	Sig.	Collinear statistics		RMSE	MAE	Adjusted R2
		B	Bootstrap mean					Tolerance	VIF			
A (210)	(Constant)	291.252	292.38	36.39	[220.26, 364.16]	6.974	.000			11.89	9.31	.571
	HR	-1.049	-1.06	0.20	[-1.44, -0.68]	-4.821	.000	0.924	1.082
	WHR	-146.572	-147.16	36.64	[-218.18, -73.38]	-3.504	.001	0.924	1.082
B (307)	(Constant)	113.049	113.16	10.52	[92.55, 133.12]	11.181	.000			13.50	11.10	0.204
	HR	-0.736	-0.74	0.13	[-0.97, -0.49]	-5.952	.000	-0.459	-0.459
C (807)	(Constant)	167.207	167.15	18.51	[131.15, 203.85]	8.869	.000			13.87	11.50	0.213
	HR	-0.705	-0.71	0.05	[-0.81, -0.60]	-12.705	.000	-0.448	-0.435
	Gender	-6.29	-6.30	1.73	[-9.63, -2.93]	-3.715	.000	-0.172	-0.14
	Height	-0.26	-0.26	0.09	[-0.45, -0.08]	-2.762	.006	0.094	-0.104
D (232)	(Constant)	106.943	106.82	13.40	[81.03, 133.30]	7.859	.000			13.53	10.58	0.344
	HR	-0.902	-0.90	0.11	[-1.11, -0.69]	-7.508	.000	-0.564	-0.54
	Total Body Water	0.639	0.64	0.23	[0.18, 1.08]	2.71	.008	0.295	0.226

Dependent variable: SDNN.

Groups: A, Obese; B, Overweight; C, Normal; D, Underweight.

RMSE, Root Mean Square Error; MAE, Mean Absolute Error.

As shown in **Table 7**, the collinearity diagnostics revealed no clear evidence of multicollinearity in the obese, overweight, or underweight groups. However, in the normal-weight group, a relatively high maximum condition index (93.035) was observed, and multiple variables exhibited substantial variance proportions within the same dimension, indicating a possible risk of collinearity. Despite this, when interpreted alongside the bootstrap validation results presented in **Table 6**, the regression coefficients for this group remained largely stable. Consequently, the overall model was deemed acceptable, although the corresponding coefficient estimates should be interpreted with caution.

**Table 7:** Collinearity diagnostics for SDNN.

Obesity group	Model	Dimension	Eigenvalue	Condition index	Variance proportion (constant)	HR	Waist-to-hip ratio	Height	Total body water
A (210)	1	1	2.991	1	0	0	0	—	—
		2	0.008	19.493	0.05	0.99	0.04	—	—
		3	0.001	51.039	0.95	0.01	0.96	—	—
B (307)	2	1	1.993	1	0	0	—	—	—
		2	0.007	17.35	1	1	—	—	—
C (807)	3	1	3.91	1	0	0	0	0	0
		2	0.079	7.044	0.01	0.01	0.35	—	0
		3	0.011	19.292	0.01	0.95	0.03	0.02	—
		4	0	93.035	0.99	0.04	0.61	0.98	—
D (232)	4	1	2.973	1	0	0	—	—	0
		2	0.022	11.502	0	0.24	—	—	0.57
		3	0.005	25.383	0.99	0.76	—	—	0.43

Dependent variable: SDNN, .

Groups: A, Obese; B, Overweight; C, Normal; D, Underweight.

RMSE, Root Mean Square Error; MAE, Mean Absolute Error; H, height; W, weight; WHR, Waist-to-hip ratio; TBW, Total body water.

**Table 8** presents the results of BMI-stratified multivariable linear regression analyses with PRV index as the outcome variable. Across all BMI categories, HR demonstrated a consistent and significant negative association with PRV index. In the obese group (Group A, n = 210), both HR (B = −0.262, 95% CI: −0.40 to −0.15, P< 0.001) and WHR (B = −25.756, 95% CI: −49.42 to −5.06, P = 0.003) were identified as independent negative predictors of PRV index. For the overweight group (Group B, n = 307), HR remained the only variable significantly associated with PRV index (B = −0.202, 95% CI: −0.269 to −0.14, P< 0.001). In the normal-weight group (Group C, n = 807), HR continued to show a negative relationship with PRV index (B = −0.160, 95% CI: −0.19 to −0.13, P< 0.001), while BMI was positively associated with PRV index (B = 0.206, 95% CI: 0.03 to 0.38, P = 0.025). Among underweight participants (Group D, n = 232), HR also exhibited a negative association with PRV index (B = −0.089, 95% CI: −0.17 to −0.02, P = 0.019), whereas skeletal muscle mass showed a significant positive association (B = 0.278, 95% CI: 0.12 to 0.44, P< 0.001).

**Table 8:** Multivariable linear regression analysis of PRV index across BMI categories among university students with 5, 000-sample bootstrap internal validation.

Groups	Variate	Non-standardized coefficient		Bootstrap SE	95% CI	t	Sig.	Collinear statistics		RMSE	MAE	Adjusted R2
		B	Bootstrap mean					Tolerance	VIF			
A (210)	(Constant)	62.037	62.592	11.426	[41.10, 85.67]	5.462	.000			3.23	2.59	.468
	HR	-0.262	-0.265	0.062	[-0.40, -0.15]	-4.433	.000	0.924	1.082
	WHR	-25.756	-26.067	11.380	[-49.42, -5.06]	-2.264	.003	0.924	1.082
B (307)	(Constant)	31.017	30.912	2.866	[25.41, 36.63]	11.786	.000			3.51	2.80	.223
	HR	-0.202	-0.201	0.034	[-0.269, -0.14]	-6.28	.000	1	1
C (807)	(Constant)	23.494	23.478	2.368	[18.74, 28.19]	10.045	.000			4.09	3.14	0.129
	HR	-0.16	-0.159	0.017	[-0.19, -0.13]	-9.978	.000	1	1
	BMI	0.206	0.205	0.091	[0.03, 0.38]	2.249	.025	1	1
D (232)	(Constant)	14.998	15.290	4.330	[7.25, 23.98]	3.973	.000			4.19	3.21	0.130
	HR	-0.089	-0.091	0.040	[-0.17, -0.02]	-2.381	.019	0.962	1.039
	skeletal muscle	0.278	0.274	0.081	[0.12, 0.44]	3.586	.000	0.962	1.039

Dependent variable: PRV index, .

Groups: A, Obese; B, Overweight; C, Normal; D, Underweight.

RMSE, Root Mean Square Error; MAE, Mean Absolute Error.

Bootstrap internal validation using 5, 000 resamples demonstrated that the estimated coefficients for all variables were highly consistent with those from original regression model. Additionally, none of the 95% confidence intervals overlapped zero, supporting the robustness and stability of the model. The adjusted R² values were 0.468, 0.223, 0.129, and 0.130, respectively. These results indicate that the model in the obese group exhibited comparatively stronger explanatory performance than the others.

As detailed in **Table 9**, the tolerance values varied between 0.924 to 1.000, while the variance inflation factors (VIFs) ranged from 1.000 to 1.082, indicating the absence of significant multicollinearity. When considered alongside the condition indices and variance decomposition proportions, these results support overall stability of the stratified regression models and confirm the reliability of the estimated parameters.

**Table 9:** Collinearity diagnostics for PRV index.

Obesity group	Model	Dimension	Eigenvalue	Condition index	Constant	HR	Waist-to-hip ratio	Body mass index	Skeletal muscle
A (210)	1	1	2.991	1.000	0.00	0.00	0.00	—	—
		2	0.008	19.493	0.05	0.99	0.04	—	—
		3	0.001	51.039	0.95	0.01	0.96	—	—
B (307)	2	1	1.993	1.000	0.00	0.00	—	—	—
		2	0.007	17.350	1.00	1.00	—	—	—
C (807)	3	1	2.986	1.000	0.00	0.00	—	0.00	—
		2	0.011	16.199	0.02	0.83	—	0.17	—
		3	0.003	33.135	0.98	0.17	—	0.83	—
D (232)	4	1	2.958	1.000	0.00	0.00	—	—	0.00
		2	0.036	9.046	0.01	0.11	—	—	0.73
		3	0.005	23.343	0.99	0.88	—	—	0.26

Dependent variable: PRV index, .

Groups: A, Obese; B, Overweight; C, Normal; D, Underweight.

RMSE, Root Mean Square Error; MAE, Mean Absolute Error; H, height; W, weight; WHR, Waist-to-hip ratio; BMI, Body mass index; SM, Skeletal muscle.

## Discussion

Drawing on data from 1, 556 university students, this study revealed that, following BMI-based stratification, individuals classified as overweight or obese demonstrated a less optimal resting autonomic profile compared with those in the normal-weight and underweight categories. This pattern was primarily reflected by higher resting heart rate, reduced SDNN, and a more pronounced decline in PRV index, particularly among obese females. Among males, SDNN values were lower in the obese group compared with the normal-weight group (48.63 ± 16.83 ms vs. 60.46 ± 15.00 ms). A similar trend was observed in females, where SDNN in the obese group was notably lower than in the normal-weight group (39.74 ± 12.52 ms vs. 55.06 ± 15.77 ms). Furthermore, obese females exhibited a significantly elevated resting HR (89.75 ± 8.71 bpm vs. 80.82 ± 9.40 bpm) alongside a reduced PRV index (10.94 ± 3.72 vs. 14.84 ± 4.44) compared with their normal-weight counterparts. In general, these results align with prior research demonstrating an association between obesity and disturbances in resting autonomic function. Increasing fat accumulation, particularly in central and visceral regions, is often associated with altered autonomic modulation, which may be reflected by elevated resting HR, reduced vagal-related variability, and decreased overall PPG-derived PRV. Collectively, these alterations contribute to a cardiovascular profile associated with elevated risk (Petretta et al., 1995; Yildiz et al., 2018; Gutin et al., 2005; Janzen et al., 2023; Tian et al., 2015; Kono et al., 2019; Lindmark et al., 2012; Verma et al., 2024; Yadav et al., 2017). This trend is further supported by review-based findings highlighting the interrelationship between obesity, visceral fat, and PRV.

Beyond the commonly reported finding that PRV is reduced in individuals with obesity, the current study indicates that, within the present experimental context, SDNN and the PRV index were more sensitive and consistent in distinguishing between groups than certain normalized frequency-domain measures. Overall, LF, HF, LFnorm, and HFnorm did not demonstrate clear or consistent differences across most BMI categories. In contrast, SDNN showed significant variation in both male and female groups, while the PRV index exhibited a more pronounced and progressive decline among females across the stratified categories. This observation implies that, within a relatively young cohort not yet presenting overt clinical disease, autonomic changes associated with obesity may initially appear as a reduction in overall sinus rhythm variability rather than a clear shift in normalized frequency-domain components. Put differently, existing evidence suggests that in the early stages of obesity, alterations in autonomic modulation are more likely to manifest as a generalized reduction in variability. Consequently, SDNN, which reflects the overall dispersion of NN intervals, and the PRV index, which captures the breadth of the variability distribution, may serve as more sensitive indicators of these early alterations. In contrast, LFnorm and HFnorm are relative indices; when both LF and HF components decrease simultaneously or are proportionally attenuated, their normalized values or ratios may remain relatively stable, thereby potentially masking early autonomic alterations. Moreover, LF-related indices and the LF/HF ratio were interpreted cautiously in the present study. These measures were not treated as direct indicators of sympathetic activity or definitive markers of sympathovagal balance, but rather as relative frequency-domain PRV parameters influenced by multiple overlapping physiological mechanisms. This interpretation aligns with established perspectives on PRV metrics: SDNN reflects overall variability across the entire recording period, whereas the PRV index, as a geometric parameter, more directly represents the overall variability pattern rather than the proportional contribution of specific frequency components (Jabbour and Iancu, 2021; Sendeski et al., 2024).

When considered alongside the body composition results, this interpretation is further reinforced within the obese subgroup. As indicated in **Table 3**, obese participants of both sexes exhibited elevated levels of BFP, WHR, VFI, and FLRI. Additionally, **Table 5** demonstrates that, within this group, SDNN showed moderate negative correlations with BFP, BMI, WHR, VFI, and FLRI, with the most consistent relationships observed for WHR, VFI, and FLRI. Together, these findings suggest that, in this cohort, the reduction in SDNN was not simply attributable to higher overall body weight, but was more strongly linked to central adiposity and the associated metabolic load. From a mechanistic standpoint, the observed decline in SDNN is unlikely to be explained by body weight in isolation. Rather, it may reflect impaired autonomic regulation arising from the combined effects of visceral adiposity, dysregulated lipid metabolism, low-grade systemic inflammation, and heightened stress reactivity. However, it is important to note that this explanation is inferential in nature, grounded in the present results and supporting literature, and does not establish a definitive causal pathway.

The more marked alterations observed in obese females warrant particular attention. In the present study, this group showed not only elevated HR and reduced SDNN, but also a clearer decline in PRV index, indicating a greater compromise in overall heart rate variability. One possible interpretation is that, during young adulthood, factors such as emotional stress reactivity, patterns of fat distribution, and hormonal influences may interact to shape resting autonomic function in female university students, thereby amplifying PRV disturbances under obese conditions. Previous research also indicates that sex-related differences in body fat composition, vagal activity, and stress-response mechanisms can influence PRV, providing physiological support for the more pronounced effects observed in females in this study (Sheema and Malipatil, 2015; Soumya et al., 2022; Mittelman et al., 2012; Mitchelmore, 2015). Although obese males also exhibited lower SDNN, several PRV-related differences were not statistically significant, implying that autonomic changes associated with obesity in young men may be less pronounced or still in an earlier phase. It is also possible that these effects are modulated by unmeasured behavioral factors. Therefore, while sex-related differences appear evident and meaningful in this study, the underlying mechanisms should be interpreted cautiously.

It is important to acknowledge that, although SDNN and PRV index demonstrated relatively strong discriminatory performance in this study, they are not entirely independent of heart rate or recording conditions. Prior research indicates that PRV measures are associated with mean HR both physiologically and mathematically; as resting HR increases, the range of NN interval variability may decrease, even when autonomic modulation remains unchanged, resulting in lower SDNN and PRV index values. Consistent with this, the BMI-stratified multivariable regression analyses in the present study revealed a stable and independent negative relationship between HR and both SDNN and PRV index across all four subgroups. This observation does not undermine the relevance of SDNN and the PRV index; instead, it highlights the need to interpret these measures within a broader framework that accounts for heart rate, autonomic function, and patterns of fat distribution simultaneously. In essence, the findings do not imply that SDNN and PRV index operate independently of HR, but rather that, under uniform resting conditions in a university population, they may more effectively capture the overall reduction in variability linked to obesity-related alterations in autonomic regulation. It should also be recognized that PRV is influenced by multiple factors, including recording duration, body position, breathing patterns, environmental conditions, and artifact correction methods. As such, conclusions about the comparative utility of different PRV metrics should be limited to the specific measurement context and analytical approach of this study, and should not be generalized without appropriate caution.

From a modeling standpoint, both the SDNN and PRV index models demonstrated strong internal consistency. As shown in **Tables 6**, **8**, bootstrap validation with 5, 000 resamples produced mean estimates for the key predictors that closely matched the original regression coefficients, with all 95% CIs remaining entirely on one side of zero, supporting the robustness of the estimates. Importantly, within the obese subgroup, the SDNN model achieved an adjusted R² of 0.571, while the PRV index model reached 0.468, both exceeding the values observed in the other BMI categories. This pattern suggests that the relationship between PRV-related measures and body fat distribution becomes more pronounced as adiposity increases to the level of obesity. Furthermore, as presented in **Tables 7**, **9**, the tolerance and VIF values across most models fell within acceptable limits, suggesting the absence of significant multicollinearity. Although the SDNN model in the normal-weight group exhibited a relatively elevated condition index, indicating a possible risk of collinearity, the bootstrap validation still demonstrated consistent coefficient directions. Collectively, these findings support the internal reliability of the models; however, some coefficients should still be interpreted with appropriate caution.

Overall, the results indicate that, among university students, especially those in the obese category, SDNN and the PRV index, both of which represent the overall variability of heart rate dynamics, may be more effective than certain normalized frequency-domain measures in detecting early autonomic alterations associated with central adiposity. This relative advantage is evident in two key ways. First, these indices demonstrated more stable and consistent relationships with BMI, WHR, VFI, and FLRI. Second, in the stratified regression models, they contributed to a more coherent explanatory framework involving variables such as HR, WHR, skeletal muscle mass, or total body water. Accordingly, the findings of this study lend support to the potential utility of SDNN and the PRV index as practical indicators for screening obesity-related cardiovascular risk in young adults, particularly in large-scale, noninvasive, and cost-effective campus health settings. However, the interpretation of underlying mechanisms should remain appropriately cautious. A more balanced conclusion is that, under the conditions of this study, these indices exhibited stronger statistical discrimination and a more coherent physiological basis for interpretation, rather than being universally superior to other PRV measures across all contexts.

## Conclusion

The findings of this study indicate that, among university students, overweight and obesity are linked to a less favorable resting autonomic profile, primarily evidenced by increased resting heart rate, decreased SDNN, and a more pronounced decline in PRV index among obese females. Compared with certain normalized frequency-domain measures, SDNN and PRV index exhibited stronger discriminatory capacity for identifying early obesity-related autonomic impairment under the conditions of this study, and they maintained more consistent associations with measures of central adiposity. These results indicate that SDNN and the PRV index may be useful as observational indicators for early cardiovascular risk assessment in obesity-related contexts among young adults. Nevertheless, their underlying mechanism and wider clinical relevance need to be further validated through longitudinal investigations and mechanistic studies.

## Limitations

This study evaluated rhythm variability using photoplethysmography (PPG) signals; therefore, the derived measures are more accurately described as pulse rate variability (PRV) rather than conventional heart rate variability (HRV) obtained from electrocardiography (ECG). In contrast to ECG, which directly captures the electrical activity of the heart, PPG reflects changes in peripheral blood volume. As a result, PRV is influenced not only by cardiac rate regulation but also by factors such as pulse transit time, vascular elasticity, microvascular dynamics, and peripheral perfusion status (Tarvainen et al., 2009; Kaufmann et al., 2011; Tarvainen et al., 2013). Previous research indicates that, when measurements are conducted under controlled conditions, such as a resting state, stable posture, and high signal quality, PPG-derived PRV can show reasonable agreement with ECG-based HRV for certain time-domain parameters, making it suitable for relative or group-level comparisons. However, the reliability of variability estimates derived from peripheral or wearable signals is highly dependent on preprocessing quality, artifact detection, and correction strategies, as even small amounts of noise or missed or extra beats can substantially distort HRV-related indices, particularly in short-term recordings (Lipponen and Tarvainen, 2019; Rogers et al., 2021; Bourdillon et al., 2022; Cosoli et al., 2021). Accordingly, PRV values are not directly equivalent to those obtained from ECG-HRV and should not be considered interchangeable measures.

This constraint may be particularly pronounced in individuals with obesity or elevated adiposity, who are more susceptible to factors such as optical signal attenuation, variations in peripheral perfusion, increased skin and subcutaneous tissue thickness, and motion-related artifacts. These influences can degrade PPG waveform quality, leading to increased variability and reduced reliability of high-frequency and other frequency-domain measures (Cosoli et al., 2021; Rogers et al., 2021; Alcantara et al., 2020). In addition, studies on wearable-derived variability and HRV preprocessing have shown that artifact burden, device type, and correction settings can substantially influence derived parameters (Saleem et al., 2022; Cutrim et al., 2024; Santos-de-Araújo et al., 2024). Available evidence further indicates that, although PPG-based PRV and ECG-derived HRV may demonstrate moderate to strong correlations in overweight adults, the level of agreement is insufficient for them to be used interchangeably. In younger populations with obesity, such as adolescents and children, acceptable agreement has been observed only for selected indices and primarily under strictly controlled conditions, for example during resting supine assessments (Cutrim et al., 2024; Santos-de-Araújo et al., 2024). Consequently, the findings of the present study should be interpreted as reflecting autonomic modulation assessed via PPG-derived PRV, rather than as direct or absolute measures of conventional ECG-based HRV.

## Data Availability

The original contributions presented in the study are included in the article/supplementary material. Further inquiries can be directed to the corresponding author.
